# Automated and manual patch clamp data of human induced pluripotent stem cell-derived dopaminergic neurons

**DOI:** 10.1038/sdata.2017.56

**Published:** 2017-04-25

**Authors:** Denise Franz, Hervør Lykke Olsen, Oliver Klink, Jan Gimsa

**Affiliations:** 1 Chair of Biophysics, University of Rostock, Rostock 18057, Germany; 2 Sophion—Biolin Scientific, Ballerup 2750, Denmark

**Keywords:** Patch clamp, Ion transport, Ion channels in the nervous system, Induced pluripotent stem cells

## Abstract

Human induced pluripotent stem cells can be differentiated into dopaminergic neurons (Dopa.4U). Dopa.4U neurons expressed voltage-gated Na_V_ and K_V_ channels and showed neuron-like spontaneous electrical activity. In automated patch clamp measurements with suspended Dopa.4U neurons, delayed rectifier K^+^ current (delayed K_V_) and rapidly inactivating A-type K^+^ current (fast K_V_) were identified. Examination of the fast K_V_ current with inhibitors yielded IC_50_ values of 0.4 mM (4-aminopyridine) and 0.1 mM (tetraethylammonium). In manual patch clamp measurements with adherent Dopa.4U neurons, fast K_V_ current could not be detected, while the delayed K_V_ current showed an IC_50_ of 2 mM for 4-aminopyridine. The Na_V_ channels in adherent and suspended Dopa.4U neurons showed IC_50_ values for tetrodotoxin of 27 and 2.9 nM, respectively. GABA-induced currents that could be observed in adherent Dopa.4U neurons could not be detected in suspended cells. Application of current pulses induced action potentials in approx. 70 % of the cells. Our results proved the feasibility of automated electrophysiological characterization of neuronal cells.

## Background & Summary

Neuronal cells are required for modeling human degenerative and genetic diseases, for example Alzheimer’s, Parkinson’s, and Huntington's diseases, as well as neurotoxic effects^1,2^. In research, the emerging alternative to primary neuronal cell cultures are neurons derived from human induced pluripotent stem cells (hiPSCs)^3^. In addition to animal models and primary animal cells, hiPSCs offer the opportunity for drug development in model systems with patient-specific physiology. Reprogramming the somatic cells of individual patients into pluripotent cells, which are capable of being differentiated into cells of all three germ layers, will be a first step toward personalized medicine. The properties of hiPSCs permit the induction of diverse neuron types and the formation of neuronal networks with predetermined parameters.

Dopaminergic neurons are major players in Parkinson’s disease. One of the main characteristics of this neurodegenerative brain disorder is the loss of dopaminergic neurons of the *substantia nigra* in the midbrain. Several animal models for Parkinson’s disease have been established to facilitate research into the mechanisms of the disease and to examine the effects of drugs on disease symptoms. While the actual dopaminergic neurons of patients remain inaccessible for experimentation, hiPSC-derived neurons allow the induced differentiation of dopaminergic neurons. Such neurons permit studies into the maintenance of the transmembrane potential^4^ and the mechanisms of deep brain stimulation^5,6^. The hiPSC-derived Dopa.4U neurons (Axiogenesis, Cologne, Germany) resemble dopaminergic neurons, i.e., the type of neurons whose regression is associated with Parkinson’s disease.

However, the establishment of a cellular disease model with Dopa.4U neurons requires electrophysiological characterization of the cells. The ‘gold standard’ for such assays is the manual patch clamp assay. This technique enables the evaluation of important phenotypic features of neuronal cells, including the examination of sodium, potassium and calcium currents, as well as transmembrane resting potentials and action potentials. To reduce the complexity and the costs of manual patch clamp investigations, automated patch clamp (APC) systems have been developed. They enable fast and highly reproducible electrophysiological examinations of suspended cells^7,8^. One example is the QPatch system by Sophion (Sophion—Biolin Scientific, Ballerup, Denmark)^8^.

Here, we present the basic electrophysiological parameters of Dopa.4U neurons obtained with the QPatch system, including the current-voltage characteristics of voltage-gated sodium and potassium ion channels as well as ligand-gated GABA receptors. Our results demonstrate the feasibility of APC characterization for neurons. Advantages and disadvantages of the APC method were evaluated by comparing our APC results with manual patch clamp measurements of Dopa.4U neurons.

## Methods

### Cell handling

Cryopreserved vials of Dopa.4U neurons were obtained from Axiogenesis (Cologne, Germany). The vials were thawed, and the neurons were cultured according to the company’s recommendations. For the APC experiments, 2.5 million Dopa.4U neurons were plated in Matrigel-coated (Corning, Wiesbaden, Germany) T12.5 cell culture flasks. Half of the Dopa.4U culture medium volume, i.e., DMEM/F12 (Life Technologies part of Thermo Fisher Scientific Inc., Waltham, MA, USA) with DA-Supplement (Axiogenesis) was changed twice a week. At 5 % CO_2_ and 37 °C, the cells could be kept in culture for more than four weeks.

To find the optimal cell harvesting process, pilot tests were performed using the manual patch clamp method. Three different detachment solutions were tested at 37 °C: Accutase® (60 min; Sigma Aldrich, Berlin, Germany), Trypsin-EDTA 1X (5 min; Pan-Biotech GmbH, Aidenbach, Germany), and Detachin (5 min; Genlantis, San Diego, CA, USA). Before applying the detachment solutions, the Dopa.4U neurons were washed with phosphate-buffered saline (PBS, without Ca^2+^/ Mg^2+^, Biochrom AG, which belongs to MERCK, Berlin, Germany). The detached cells were spun down at 200×g for 5 min, washed with the external bath solution for manual patch clamp measurements (see below) and then centrifuged again under the same conditions. To test the influence of the detachment solutions on the gigaseal and the success rate, manual patch clamp analysis was performed in petri dishes after the sedimentation of the cells.

Automated electrophysiological measurements were obtained with the QPatch system (Sophion—Biolin Scientific, Ballerup, Denmark) 10 days after seeding (10 DIV; please note that 'DIV' is commonly used for 'days *in vitro*'). After harvesting, the cells were centrifuged and resuspended in QPatch-external bath solution (see below). The automated QPatch system with the integrated centrifuge permits the direct transfer of the cell suspension without a second washing step. Reference measurements were conducted with a manual patch clamp system. For this, 0.1 million Dopa.4U neurons were cultivated on Geltrex-coated (Gibco part of Thermo Fisher Scientific Inc., Waltham, MA, USA) 12-mm round coverslips (Menzel GmbH, Braunschweig, Germany) in 24-well plates. First, experiments were initiated after the cells had been carefully washed with external bath solution for manual measurements, in order to remove proteins from the culture medium. Then, the coverslip with the Dopa.4U neurons was transferred to the recording chamber (RC-25; Hugo Sachs Elektronik—Harvard Apparatus GmbH, March-Hugstetten, Germany) and covered with external bath solution. To permit the correlation of the ion channel composition with the degree of maturity of the Dopa.4U neurons, the measurements were obtained at different points in time. Neuronal maturation, which is correlated with the progressive expression of ion channels, was electrophysiologically detected by increasing ion channel current magnitudes. Cells with current magnitudes higher than 1 nA were considered mature.

### Patch clamp solutions

The external bath solution for the QPatch system contained 2 mM CaCl_2_, 1 mM MgCl_2_, 10 mM HEPES, 4 mM KCl, 145 mM NaCl, and 10 mM glucose at pH 7.4 and 305 mOsmol/kg. The internal solution contained 120 mM KF, 20 mM KCl, 10 mM HEPES, and 10 mM EGTA at pH 7.2 and 300 mOsmol/kg. The different ion compositions of the solutions resulted in a liquid junction potential of 7 mV, which had to be subtracted when interpreting the automated measurements. The external bath solution for manual measurements contained 137 mM NaCl, 5 mM KCl, 3 mM CaCl_2_, 0.1 mM MgCl_2_, 10 mM glucose, 0.01 mM glycine, and 5 mM HEPES at pH 7.3 and 320 mOsmol/kg. The intracellular solution contained 140 mM potassium-D-gluconate, 5 mM KCl, 0.5 mM CaCl_2_, 0.5 mM MgCl_2_, 5 mM EGTA, 10 mM HEPES, and 2 mM K_2_-ATP at pH 7.2 and 315 mOsmol/kg. In the manual measurements, the liquid junction potential was 15 mV.

For the APC experiments, stock solutions of 1 mM tetrodotoxin (TTX; Alomone labs, Jerusalem, Israel) and 1 mM γ-aminobutyric acid (GABA; Sigma Aldrich) were prepared in dimethyl sulfoxide (DMSO; Sigma Aldrich). The desired concentrations of the solutions with tetraethylammonium chloride (TEA; Sigma Aldrich) and 4-aminopyridine (4-AP; Sigma Aldrich) were freshly prepared in QPatch external bath solution before each experiment. For the manual measurements, stock solutions of 1 mM TTX (Tocris Bioscience, Bristol, United Kingdom) and 100 mM GABA were prepared with the external bath solution while 1 M 4-AP and 1 M 3,4-aminopyridine (3,4-AP; Sigma Aldrich) were dissolved in DMSO, respectively.

### Recordings

APC experiments were conducted with the QPlate 48 measuring interface (Sophion) with 48 single holes. Gigaseals were formed by mild suction after the Dopa.4U neurons had been positioned at the holes. The Dopa.4U neurons were characterized in whole-cell mode, which was achieved in two different ways. Transition to whole-cell mode was achieved either by suction-pulse steps or by patch perforation by supplementing the internal solution of the QPatch with 10 μM β-escin (Sigma Aldrich) before the experiment. In the latter method, the suction pulses could be omitted. In whole-cell mode, the internal cell potential was clamped to −60 mV.

In the first test for the presence of voltage-gated ion channels, a voltage step protocol was used with progressive 500-ms wide, 10-mV square-shaped steps in the range of −100 to +60 mV. Between each step, the cell was clamped to −100 mV for 200 ms in order to inactivate the ion channels. For the pharmacological analysis of the three specific ion channel types detected, single voltage pulses were applied. While the two outward-rectifier ion channel types were examined with +40-mV pulses, the inward rectifier ion channel type was examined with 0-mV pulses. To induce action potentials, a current clamp protocol with 10-s, 20-pA square-shaped steps was used in the 20 to 60 pA range. In GABA-gated channel experiments, the GABA-induced current was recorded while the cells were clamped at −60 mV. Evaluation of all the QPatch data was performed with the device-specific software (Assay Software Version 5.2, Sophion).

The manual patch clamp system for the reference measurements featured a triple EPC 10 amplifier with PatchMaster software (both HEKA Elektronik, Lambrecht/Pfalz, Germany). The recording chamber with the Dopa.4U neurons on a coverslip was connected to an ALA-VM8 perfusion system (ALA Scientific Instruments, New York). Borosilicate glass patch pipettes (GC150F-10; Harvard Apparatus, Kent, United Kingdom) with resistances of 3–4 MOhm were used. In the whole-cell configuration achieved by suction-pulse steps, a holding potential of −60 mV was applied. Voltage-gated ion channels were characterized with the automated step protocol with the +5-mV steps protocol. The +5-mV step protocol was also used to test the effects of the compounds. The GABA-induced currents were investigated by filling the measuring volume around the cell with GABA medium, using the ALA-VM8 and the simultaneous recording of the current response. In the manual patch clamp system, action potentials were induced by applying 500-ms, +40-pA square-shaped pulses. All the automated and manual experiments were performed at room temperature (23 °C).

### Code availability

All QPatch and manual patch clamp raw data are openly accessible (see Data Records). The Sophion QPatch Assay software application (version 5.2) is required to edit the QPatch raw data. The manual patch clamp raw data can be edited with Excel. Representative QPatch and manual patch clamp data were converted into Excel files for easy editing (see Usage Notes).

For the manual determination of the Na_V_ channel activation curve, only data of adherent cells with serial resistances below 30 MOhm were chosen. The obtained raw data were edited in Excel for the offset-corrections. The current values were corrected after linear regression. The voltage values were corrected by the liquid junction potentials. After these corrections, the activation kinetics were fitted by:

(1)gNa=INaE−Erev

with *g*_*Na*_, *I*_*Na*_, *E*, and *E*_*rev*_ being the conductivity of Na_V_ channel, the maximum current amplitude of the Na_V_ channel, the voltage step amplitude, and the read offs of the reversal potentials from the I-V relationships, respectively.

For the dose-response curves, the means of the maximum current values, as well as the standard error of the means at the corresponding compound concentrations were calculated with Excel. The results were transferred to Origin Lab to create the dose-response curves. The dose responses were fitted with the sigmoidal function:

(2)y=A1+A2−A11+10(logx0−x)p

with *A*1, *A*2, log_*x*0_, and *p* being the bottom and top asymptotes, the center point, and the Hill slope, respectively.

## Data Records

Raw data on automated QPatch and manual patch clamp experiments are available at *figshare* (Data Citation 1). The *QPatch raw* folder contains all the project files (Nyborg_project568 to Nyborg_project849), as well as the *Falster projects* folder that contains all the assay protocol files used. Our data analysis is documented in the *statistics Dopa.4U QPatch* and *statistics pharmacological research QPatch* Excel files.

Raw data on manual patch clamp measurements are stored under *manual patch clamp raw.* The first *detachment tests* subfolder contains data on the experiments with the three Accutase, Trypsin, and Detachin solutions. The second *pharmacology* subfolder contains the raw data on the pharmacological measurements. The results are documented in the *statistics Dopa.4U manual patch clamp, statistics activation curve* and *statistics pharmacological research manual patch clamp* Excel files.

## Technical Validation

**Figure 1:**
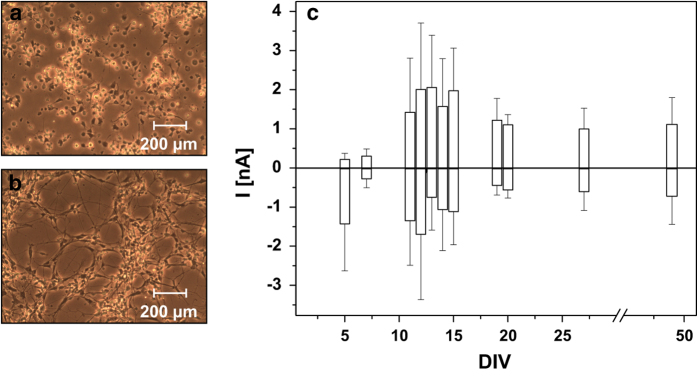
Morphological and electrophysiological maturation of Dopa.4U neurons. Dopa.4U neurons on Geltrex-coated cell culture dishes 1 h (**a**) and 24 h (**b**) after seeding. Neurons started spreading extensions in (**a**), forming a network in (**b**). To identify the optimal measuring period, the maximum amplitudes (*n*>3) of the delayed K_V_ (positive values) and the inward Na_V_ (negative values) currents were plotted over DIV (**c**).

One hour after seeding, the Dopa.4U neurons began spreading neurites. A neuron-like network developed within 24 h (Fig. 1a,b). To determine the degree of maturation of the cells electrophysiologically, current-voltage (I-V) relationships of adherent Dopa.4U neurons were manually recorded between DIV 5 and DIV 49. Inwardly and outwardly directed currents of different magnitudes were detected on these days. The results are presented in Fig. 1c. Because amplitudes in the range of 1 to 2 nA could only be reliably detected between DIV 10 and DIV 20, we considered this period optimal for further ion channel characterizations.

**Figure 2:**
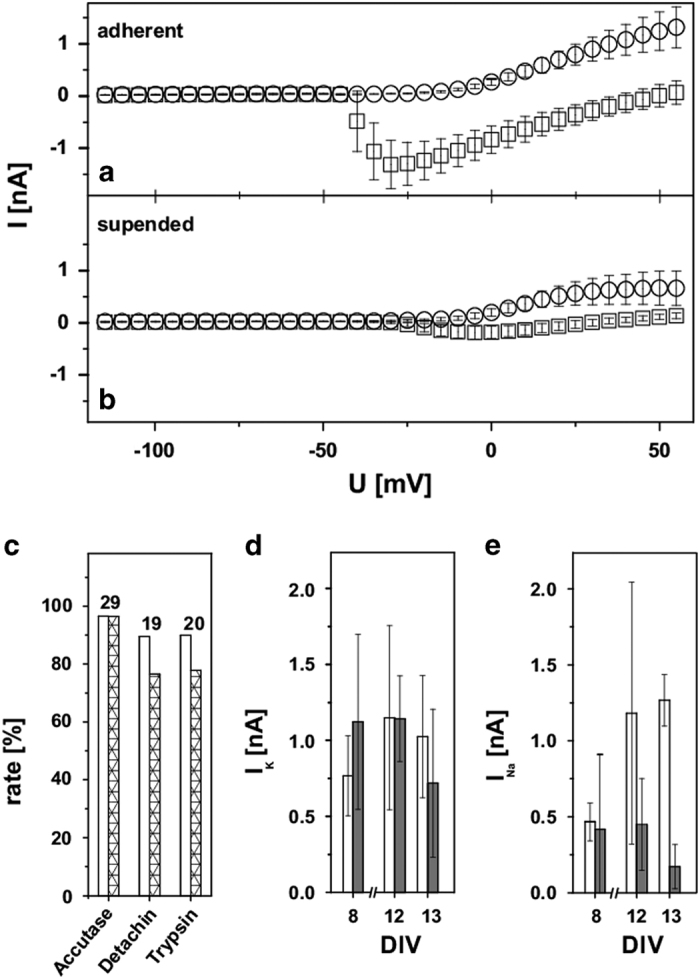
Electrophysiological characteristics of adherent and suspended Dopa. 4U neurons. Manual measurements of ion channel properties applying progressive 500-ms wide 5-mV square-shaped steps from −100 mV to 60 mV. I-V relationship of Na_V_ and delayed K_V_ currents for adherent (**a**) compared to suspended (**b**) Dopa.4U neurons. Please note the −15-mV abscissa shift, resulting from the liquid junction potential. Measurements indicated that suspended and adherent cells had the same ion channel compositions, but with altered current magnitudes. (**c**) Effects of Accutase, Detachin or Trypsin-EDTA 1X treatment on the gigaseal rate (white columns) and on the success rate (gigasealed cells with both, delayed K_V_ and Na_V_ currents; striped columns). The number of experiments is displayed above the bars. (**d**,**e**) Magnitudes of the delayed K_V_ and Na_V_ currents for the adherent (white columns) and suspended (gray columns) cells before and after harvesting at DIV 8 (n_adherent_=3, n_suspended_=9), 12 (n_adherent_=3, n_suspended_=10), or 13 (n_adherent_=4, n_suspended_=9). All error bars are defined as s.e.m.

The data from the detailed manual investigations of the Na_V_ and K_V_ channels are shown in Fig. 2. Figure 2a shows I-V relationships for gigasealed adherent Dopa.4U neurons, while Fig. 2b presents the results of the suspended neurons after detachment with Accutase. Accutase treated cells showed the best gigaseal and success rates of the three different detachment solutions, Accutase, Detachin, and trypsin-EDTA 1 X that were tested for cell harvesting (Fig. 2c). A successful experiment was characterized by the successful detection of both K^+^ and Na^+^ currents in the same gigasealed cell. In successful experiments, the serial resistances were below 50 MOhm and remained stable over the whole measuring periods. With Accutase, the gigaseal rate was up to 97 %, of which 96 % could be successfully measured. In contrast, Detachin harvesting resulted in 89 % gigaseal rate and 76% success rate, while Trypsin-EDTA 1X resulted in 90% gigaseal rate and 78 % success rate. Because of these results, Accutase was implemented for the automated QPatch tests. After test runs with 1 h of Accutase treatment as recommended by Axiogenesis (‘Detachment of neurons from T-flask’), the incubation period was reduced to 15 min according to a recommendation by Sophion without detectable differences in the experimental outcome.

As discussed above for the manual patch clamp, the Dopa.4U neurons showed a notable sealing behavior. After improvements in the initial pressure protocol of the QPatch system, a gigaseal rate of up to 90 % could be achieved once a cell had been attached to a QPlate hole. Experiments were considered successful and complete when the serial resistances in the whole-cell configuration were below 15 MOhm and the membrane resistances were higher than 100 MOhm. After parameter optimization, success rates of up to 70% were achieved. Alternatively, we examined the effect of the membrane-perforating substance escin on the quality of the whole-cell access. In our experiments, escin did not improve the gigaseal and success rates (data not shown). Of the 48% of the Dopa.4U neurons with a gigaseal, 52% were successfully measured in the presence of escin in the internal solution of the QPatch system (*n*=19).

Comparison of the manually measured I-V relationships of the suspended and adherent Dopa.4U neurons indicated the presence of the same channels. However, the suspended cells presented with reduced amplitudes. Figure 2d,e show the difference in the maximum current amplitudes of the delayed K_V_ and Na_V_ channels between adherent and Accutase-treated suspended Dopa.4U neurons for different DIV. Our measurements suggested that the potassium currents had larger amplitudes for the suspended cells (1.1±0.6 nA) than for the adherent cells (0.8±0.3 nA) at DIV 8 (Fig. 2d). While the difference largely disappeared at DIV 12 (suspended cells: 1.1±0.3 nA; adherent cells: 1.2±0.6 nA), the initial relation seemed to be inverted (suspended cells: 0.7±0.5 nA; adherent cells: 1±0.4 nA) at DIV 13. With the sodium currents, the effects were more pronounced (Fig. 2e). The current amplitudes of adherent (0.4±0.1 nA) and suspended cells (0.4±0.5 nA) did not differ at DIV 8. While the current magnitudes of the adherent cells increased (DIV 12: 1.2±0.9 nA, DIV 13: 1.3±0.2 nA), the magnitudes of the suspended cells decreased with time (DIV 12: 0.4±0.3 nA; DIV 13: 0.2±0.1 nA), resulting in current magnitudes for the adherent cells, which were three times higher than for the suspended cells. We assume that the effects of the cell harvesting process were more adverse for matured cells than for juvenile cells and have a stronger impact on the state of the sodium channel than on that of the potassium channels.

The current magnitudes in the manual pretest, which were generally higher than 0.5 nA, indicated the feasibility of electrophysiological studies with the automated patch clamp system. In the automated measurements, up to 90% of the cells were successfully giga-sealed, most of them (approx. 94%) with detectable currents. While two voltage-gated ion channel types (delayed K_V_ and Na_V_ channels) were identified with the manual system, an additional third type (fast K_V_) could be detected with the QPatch system (Table 1). This third type was characterized by a rapidly inactivating A-type K^+^ current and was found in most of the cells alone or in combination with the other two channel types. Note that it cannot be fully excluded that a reorganization or damage to the potassium channel subunits due to the harvesting process and the different cell handling steps may have caused the differences observed in the current behavior of the adherent and suspended cells.

**Figure 3:**
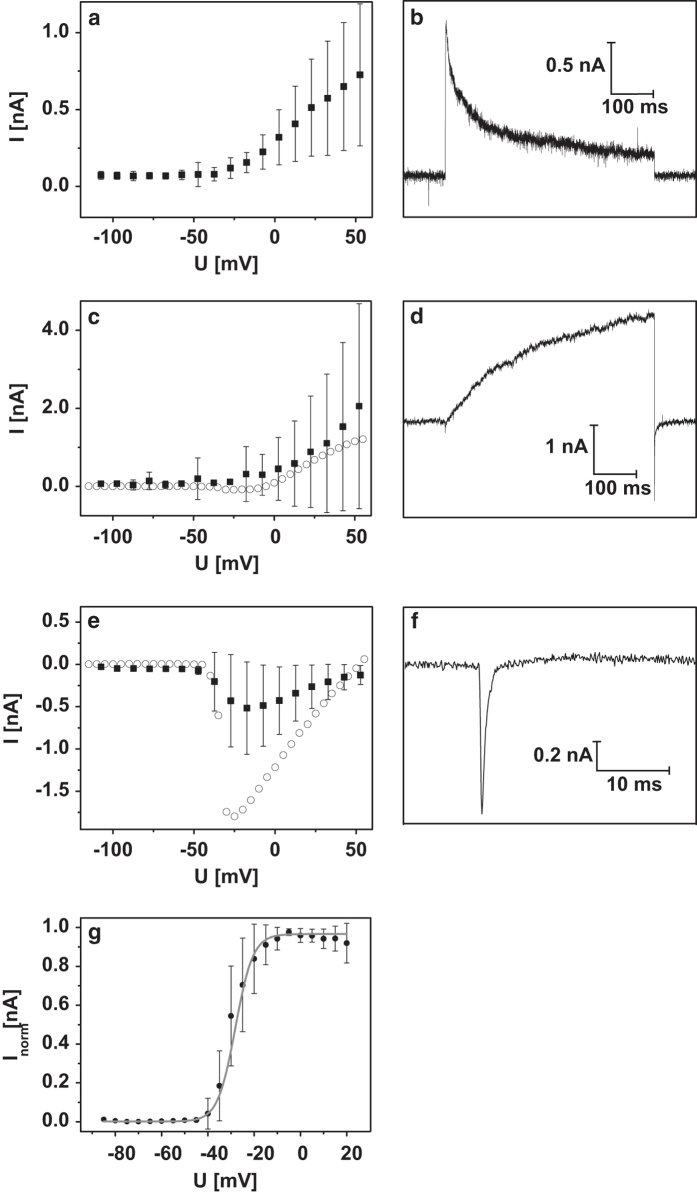
Automated patch clamp experiments. In QPatch measurements, three different ion currents could be detected in suspended Dopa.4U neurons. The appropriate I-V relationships are shown in (**a**,**c**,**e**). Please note the −7-mV abscissa shift in the mentioned I-V relationships resulted from the liquid junction potential. (**a**) The I-V relationship of the fast K_V_ indicates channel activation at −32±7 mV (*n*=204). (**b**) The appropriate raw current trace for the fast K_V_ channel at a voltage step of +60 mV. (**c**) The I-V relationship of the delayed K_V_ indicates channel activation at −27±14 mV (*n*=45), which is similar to the results of the manual patch clamp measurements (the dotted line represents a typical single cell). (**d**) The appropriate raw current trace for the delayed K_V_ channel at a voltage step of +60 mV. (**e**) The Na_V_ channel was activated at −37±8 mV (*n*=106), which is similar to the results of the manual measurements (the dotted line represents a typical single cell). (**f**) The appropriate raw current trace for Na_V_ currents at −10 mV. From the manual measurements, a half activation potential of V_0.5_=−28 mV (*n*=9) was obtained (**g**). All error bars are defined as s.e.m.

Figure 3 presents details of the three different ion channel types obtained with the automated patch clamp method. The obtained parameters were compared with the manual results and are summarized in Table 2. An inactivation time of 46 ms was determined for the fast K_V_ channel when setting the current decay at the maximum voltage of +60 mV (Fig. 3a,b). This corresponds to the 47 ms value for the K_V_ 1.4 channel type reported in the literature^9^. In our experiments, the magnitude of the fast K_V_ current increased proportionally with the increasing step voltages, unlike the maximum-current plateau behavior commonly observed in these channels. We presume that the presence of two-pore-domain potassium (K_2P_) channels in the Dopa.4U neurons is the likely reason for this ohmic behavior^10^. This behavior did not permit the calculation of a half-activation potential.

The parameters of the delayed K_V_ channels detected with the automated and manual setups were similar (Fig. 3c). Additionally, for the delayed K_V_ channel, a half-activation potential could not be determined because of a continuously increasing current for the increasing step voltages, which could also be elicited by the presence of the K_2P_ channels. At the maximum step voltage of +60 mV, the activation time was 980 ms with an inactivation time of 3.8 ms for the tail current (Fig. 3d).

The presence of Na_V_ channels in the Dopa.4U neurons is characteristic of neuronal cells. In our experiments, their properties were very similar when detected with the automated and manual methods (Fig. 3e,f). The same opening voltages were detected with both methods (Table 2), while the automated method was more suitable for the determination of the inactivation time. For the maximum current seen at −17 mV, an inactivation time of 0.5 ms was detected (Fig. 3f). We calculated the half-activation potential from the manual measurements because it allowed us to check the cell status optically and choose only the cells with even morphology (Fig. 3g).

**Figure 4:**
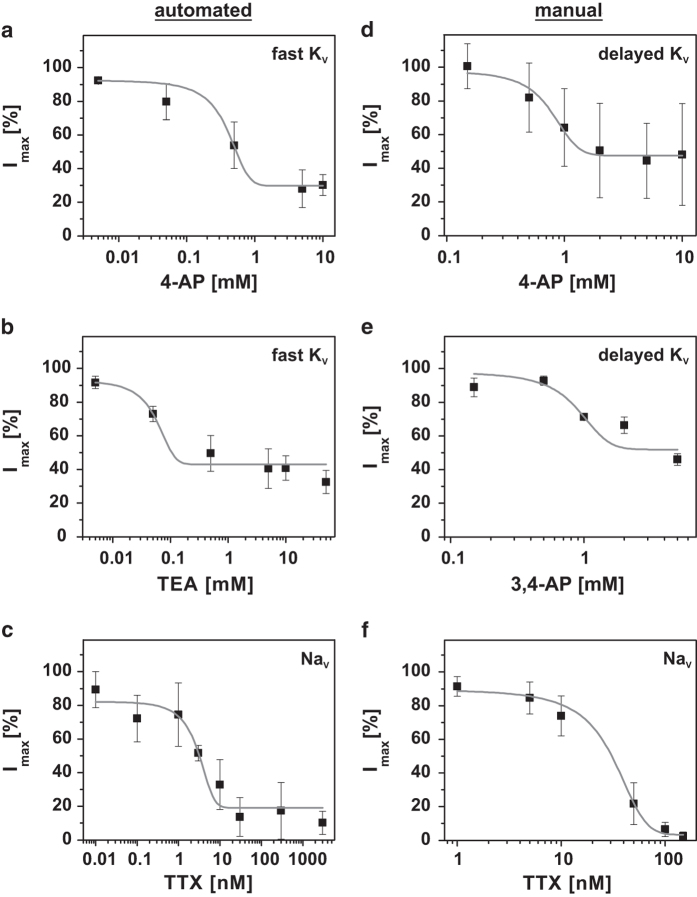
Pharmacological studies with the automated setups (a–c) and manual (d–f). The fast K_V_ channel was examined with 4-AP (**a**) and TEA (**b**) showing IC_50_ values of 0.4±1.3 mM (*n*=10) and 0.1±0.04 mM (*n*=7), respectively. The delayed K_V_ channel was examined with 4-AP (**d**) and 3,4-AP (**e**) showing IC_50_ values of 1±0.5 mM (*n*=9) and 0.9±0.75 mM (*n*=3), respectively. The Na_V_ channel was examined with TTX in the automated (**c**) and manual (**f**) patch clamp measurements yielding IC_50_ values of 2.9±0.01 nM (*n*=9) and 27±10 nM (*n*=4), respectively. All error bars are defined as s.e.m.

For a pharmacological classification of the ion channels, the typical blockers 4-AP, 3,4-AP, TEA, and TTX were used (cf. Fig. 4 and Table 2). From measurements at four or more different concentrations, dose-response curves of single cells were obtained. Figure 4a,b presents QPatch measurements of the fast K_V_ channel after 4-AP and TEA treatment. Together with our determination of the inactivation time, the IC_50_ value of 4-AP treatment confirms the existence of a K_V_ 1.4 subtype predicted by Caggiano *et al.*^11^. Nevertheless, even at their highest concentrations, neither of the two compounds blocked 100 % of the outward directed current, neither from the fast K_V_ nor from the delayed K_V_ channels (Fig. 4d,e).

For the delayed K_V_ channels, their low incidence in Dopa.4U neurons did not produce sufficient data for the calculation of dose-response curves in the automated experiments (Table 1). Therefore, only the manually detected results were used for the pharmacological analyses. Because of the huge discrepancies in the results with 4-AP (Fig. 4d), we also tested the more selective (although still subtype-unspecific) potassium channel blocker 3,4-AP (Fig. 4e). With this compound, scatter was reduced, suggesting a more selective delayed K_V_ block. Nevertheless, a complete block was not possible and potentially suggests another unknown outwardly directed current, for example, mediated by the K_2P_ channels that are not affected by common blockers.

When TTX was used to identify the subtype of the sodium channel, different IC_50_ values were obtained in the manual and automated measurements (cf. Fig. 4f,c, and Table 2). These differences suggest that two or more sodium channel subtypes (or modifications of one or more subtypes during the harvesting process) are present in the Dopa.4U neurons, for example, the Na_V_ 1.1 to Na_V_ 1.4 subtypes^12^. We believe that the Na_V_ 1.5 through Na_V_ 1.9 subtypes can be excluded because of higher half-activation potentials, higher or lower inactivation times, and higher TTX concentrations required for their inactivation.

In addition to the voltage-gated ion channels, ligand-gated GABA receptors were investigated. Measurements with the manual patch clamp indicated a response of the adherent Dopa.4U neurons to 10 μM GABA. From DIV 13 on, small GABA currents (ΔI=10 pA, data not shown) could be detected. Experiments at different DIV showed a wide range for the GABA-induced current amplitudes with currents that were rarely higher than 1 nA. In the experiments, we could not establish a relationship between the degree of maturation and current magnitude.

However, GABA-induced currents could never be detected in suspended cells, neither with the manual nor with the automated setup, even though different GABA concentrations (10 and 100 μM) were tested at DIV 14 and DIV 27. A possible reason for the absence of GABA currents could be the loss of GABA receptors during our harvesting process. However, Haythornthwaite *et al.*^13^ showed that GABA-induced currents are, in principle, detectable with the automated patch clamp system.

**Figure 5:**
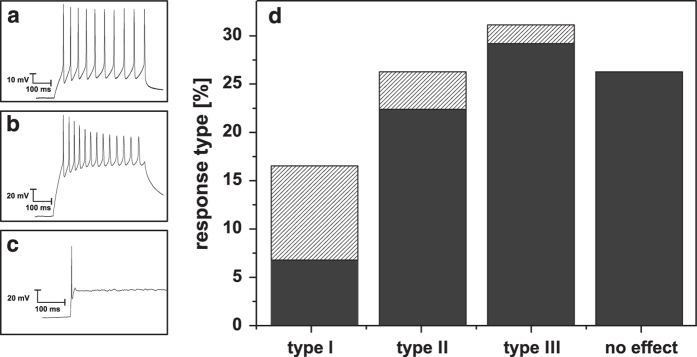
Electrical stimulation. The application of 500-ms wide 40-pA square-shaped pulses induced three types of action potential responses (**a**–**c**). In total 103 cells were measured, yielding the distribution in (**d**) (The striped parts of the columns represent live-shipped neurons). Type I (**a**) shows action potentials with constant amplitude over the whole period of stimulation. Type II (**b**) represents decreasing action potentials during the pulse duration. Type III (**c**) shows only an initial action potential at the beginning of the current pulse application. No effects were observed in 26 % of the attempts. Type III could be detected on a suspended Dopa.4U neuron measured with the QPatch by applying 10-s wide 20 pA square-shaped steps in the range of 20 pA to 60 pA (**c**).

Finally, we tested the electric excitability of the Dopa.4U neurons. The manual patch clamp setup allowed for the induction of action potentials in single Dopa.4U neurons by the application of 500-ms wide 40-pA square-shaped pulses. Three different response types were observed in the induced action potentials (*n*=103; Fig. 5a–d). Type I, observed in 16 % of the cells was characterized by action potential trains of constant amplitudes during the time of pulse application (Fig. 5a). Type II, observed in 26 % of the cells was characterized by trains with amplitudes decreasing during the time of pulse application (Fig. 5b). Type III, observed in 31 % of the cells was characterized by a single action potential peak immediately after pulse application (Fig. 5c). No effect was observed in 26 % of the attempts.

Hartfield *et al.*^3^, Tepper *et al.*^14^ and other groups described changing response patterns during cell maturation. In these experiments, matured dopaminergic neurons showed typical pace-making spike trains. We did not observe maturity-dependent effects in the Dopa.4U neurons. The type I response frequently occurred at early DIV of Dopa.4U neurons that were live-shipped, while the type III response was more often found in cultures of cryopreserved Dopa.4U neurons.

Action potentials could also be induced in QPatch experiments. Figure 5c shows type III responses of the same single cell to five consecutive pulses.

## Usage Notes

The Sophion QPatch Assay software application (version 5.2) is required to edit the QPatch raw data. This software application was also used for data analysis, e.g., for determining the dose-response curves and IC_50_ values. Alternatively, the edited raw data can be transferred into Excel sheets for further analysis (see Data Records section).

For documentation of the manual patch clamp raw data, *.dat files, which can be edited with Excel, were used. The files also contain examples of data-processing workflows. For pharmacological analysis, the data were transferred into Origin sheets in order to use the dose-response fit functions provided in the Origin software package (v8.1.13.88, non-commercial, www.OriginLab.com).

## Additional Information

**How to cite this article:** Franz, D. *et al.* Automated and manual patch clamp data of human induced pluripotent stem cell-derived dopaminergic neurons. *Sci. Data* 4:170056 doi: 10.1038/sdata.2017.56 (2017).

**Publisher’s note:** Springer Nature remains neutral with regard to jurisdictional claims in published maps and institutional affiliations.

## Supplementary Material



## Figures and Tables

**Table 1:** Abundance of cells with certain ion channel type combinations among all cells with detectable currents in the QPatch measurements.

**Abundance of cells [%]**	**Na**_**V**_	**Fast K**_**V**_	**Delayed K**_**V**_
0.4	+		
32.4		+	
16.2			+
26.3	+	+	
2.4	+		+
6.9		+	+
15.0	+	+	+
Abundance of channel types [%]	44.1	80.1	40.5
The last line presents the total abundance of the three ion channel types.			

**Table 2:** Comparison of the results obtained by the manual and automatic measurements of adherent and suspended Dopa.4U neurons.

	**Manual patch clamp adherent Dopa.4U**	**Automated patch clamp suspended Dopa.4U**
*Effective system properties*		
gigaseal rate	up to 97 %	up to 90 %
success rate	up to 96 % of the gigasealed cells with both, delayed K_V_ and Na_V_ currents	up to 70 % of the gigasealed cells with serial resistances ≤15 MOhm and membrane resistances ≥100 MOhm throughout the whole experiment
successful experiments per day	up to 20	up to 384
*Ion channel characteristics*		
**fast K**_**V**_	—	U_open_=−32±7 mV (*n*=204)
4-AP	—	IC_50_=0.4±1.3 mM (*n*=10)
TEA	—	IC_50_=0.1±0.04 mM (*n*=7)
**delayed K**_**V**_	U_open_=−16±4 mV	U_open_=−27±14 mV (*n*=45)
4-AP	IC_50_=1±0.5 mM (*n*=9)	—
3,4-AP	IC_50_=0.9±0.75 mM (*n*=3)	—
**Na**_**V**_	U_open_=−39±7 mV	U_open_=−37±8 mV (*n*=106)
	V_0.5_=−28 mV	—
TTX	IC_50_=27±10 nM (*n*=4)	IC_50_=2.9±0.01 nM (*n*=9)
**GABA**	detectable	not detectable
U_open_, voltage for channel activation.		
V_0.5_, half-activation potential.		
